# 

**Published:** 1913-12-27

**Authors:** 


### CONTENTS.

| | PAGE |
|---|---|
| **The Centre of Christmas Blessedness** ... ... | 327 |
| **The Business of Charity: Remarkable Examples** ... ... ... ... ... ... | 328 |
| **Hospital and Institutional News** ... ... ... | 329 |

The King's Hospital Presents—The High Average of Prosperity—Hurry Up Institutions!—The Dinner to M. Anatole France—First Complaint Against the New "King's"—The Condition of Cardiff Workhouse—The New Bury Infirmary—The Coming Poor-Law Conference—Eastes *v.* Russ: Result of Appeal—Unqualified Practitioners and their Fortunes—Tuberculosis Dispensaries for London—Hospital Profits by Industrial Dispute—Financial Mis-management at Devonport—Christmas Advertising—Margarine in Infirmaries—Research Work at the Brompton Hospital—Anti-Phthisis Convalescent Homes—A New Infirmary for Lambeth?—The Punishment of Panel Doctors—The Beit Research Fellowships—The New Year's Reference Books—Medical Travelling Past and Present—Grants to London Tuberculosis Dispensaries—Doctor's Bequest to St. Bartholomew's Hospital.

| | PAGE |
|---|---|
| **Why this World is Like a Hospital** ... ... | 335 |
| By the Rev. FATHER BERNARD VAUGHAN, S.J. | |
| **The Management of St. George's Hospital** ... | 337 |
| Special Court Negatives the Proposed Changes. | |
| Text of the Amendment. | |
| Criticism and Interruptions. | |
| The Questions of Amalgamation and Site. | |
| An Independent Committee to Select a Site. | |
| **My Impression of the Hospital** ... ... ... | 340 |
| The Unaided Composition of a Patient aged Eleven. | |

### CONTENTS—(*continued*).

PAGE

**The League of Mercy: Annual Meeting at St. James's Palace** ... ... ... ... ... ... 341
Prince Alexander of Teck's Address.
The Results in 1913.
How to Raise £50,000.
The Triumph of the Voluntary System.

**The Metropolitan Hospital Sunday Fund** ... 346
The Discussion at the Annual Meeting.
A Serious Decrease in Income.
Don't Grow Weary but Work for the Hospitals.

**The Editor's Letter-Box** ... ... ... ... ... 348

The Elimination of the Middle Classes.

**Appointments** ... ... ... ... .. ... 348

**Gifts and Bequests** ... ... ... ... ... ... 348

**The Profession of Medicine** ... ... ... 349

What it Has Come to in Wales.

**The Hammersmith Meeting: An Impracticable Programme** ... ... ... ... ... 350

**National Medical Union Notes** ... ... ... 350

**The National Medical Guild** ... ... ... 351

The Meeting of Practitioners at Hammersmith.

The Coming Crisis of 1915.

Need for a Safe Fighting Fund.

The Position of Medical M.P.s.

The Marquis of Zetland *v.* the B.M.A.

What Strength has the Guild?

